# psychiatric manifestations of corticosteroid therapy

**DOI:** 10.1192/j.eurpsy.2023.1575

**Published:** 2023-07-19

**Authors:** Z. El Maataoui, H. B.

**Affiliations:** Hôpital AR-RAZI, salé, Morocco

## Abstract

**Introduction:**

Children and adolescents treated with corticosteroids (CS) may experience psychiatric side effects, including psychotic symptoms. These can occur at any time during treatment, including withdrawal. There is evidence in the adult literature that higher doses of CS increase this risk. However, the dose-response relationship is not clearly identifiable. This is probably a reflection of the complexity of the effects of CS on the central nervous system and the body.

**Objectives:**

the objective of this study is to discuss the psychiatric manifestations secondary to corticosteroid therapy

**Methods:**

This is a descriptive study of 10 children with a history of somatic pathologies for which they were placed on oral, nasal, or intravenous corticosteroid therapy and who during the course of this corticosteroid therapy presented with various psychiatric manifestations.

**Results:**

The majority of the children studied were male, i.e., 7 boys to 3 girls, six children either 60%, were on nasal corticosteroids; 20% on intravenous corticosteroids, ., 20% orally.

The psychiatric manifestations noted were represented by : depressive disorder in 8 childrens Anxiety in 7 childrens a psychotic disorder in one child ADHD in 3 childrens.

**Conclusions:**

A causal role of corticosteroid therapy in the development of mental disorders in children and adolescents has been widely discussed but the results are controversial with respect to the route of administration, the relationship with the dose and the chronology of the development of mental disorders.

**Disclosure of Interest:**

None Declared

